# Accumulation of geranylgeranylated chlorophylls in the pigment-protein complexes of *Arabidopsis thaliana* acclimated to green light: effects on the organization of light-harvesting complex II and photosystem II functions

**DOI:** 10.1007/s11120-021-00827-1

**Published:** 2021-05-04

**Authors:** Václav Karlický, Zuzana Kmecová Materová, Irena Kurasová, Jakub Nezval, Michal Štroch, Győző Garab, Vladimír Špunda

**Affiliations:** 1 Department of Physics, Faculty of Science, University of Ostrava, Chittussiho 10, 710 00 Ostrava, Czech Republic; 2 Global Change Research Institute, Czech Academy of Sciences, Bělidla 986/4a, 603 00 Brno, Czech Republic; 3 Biological Research Center, Institute of Plant Biology, Temesvári körút 62, 6726 Szeged, Hungary

**Keywords:** *Arabidopsis thaliana*, Chlorophylls, Green light, Thylakoid membrane, Structure and function of photosystem II, Thermal stability

## Abstract

Light quality significantly influences plant metabolism, growth and development. Recently, we have demonstrated that leaves of barley and other plant species grown under monochromatic green light (500–590 nm) accumulated a large pool of chlorophyll *a* (Chl *a*) intermediates with incomplete hydrogenation of their phytyl chains. In this work, we studied accumulation of these geranylgeranylated Chls *a* and *b* in pigment-protein complexes (PPCs) of *Arabidopsis* plants acclimated to green light and their structural–functional consequences on the photosynthetic apparatus. We found that geranylgeranylated Chls are present in all major PPCs, although their presence was more pronounced in light-harvesting complex II (LHCII) and less prominent in supercomplexes of photosystem II (PSII). Accumulation of geranylgeranylated Chls hampered the formation of PSII and PSI super- and megacomplexes in the thylakoid membranes as well as their assembly into chiral macrodomains; it also lowered the temperature stability of the PPCs, especially that of LHCII trimers, which led to their monomerization and an anomaly in the photoprotective mechanism of non-photochemical quenching. Role of geranylgeranylated Chls in adverse effects on photosynthetic apparatus of plants acclimated to green light is discussed.

**Supplementary Information:**

The online version contains supplementary material available at 10.1007/s11120-021-00827-1.

## Introduction

Spectral quality of photosynthetic active radiation together with its intensity significantly influence plant metabolism, growth and development. Green light is still associated with the misconception that it is only poorly absorbed by plant leaves in contrast to blue and red light. While isolated photosynthetic pigments absorb very weakly in the 500–600 nm range, green light is absorbed much more by intact leaves than in solution due to light scattering in plant tissue (DeLucia et al. 1996) and the flattening effect causing attenuation of strongly absorbed blue and red light, but only marginal decrease of weakly absorbing green light (Terashima et al. 2009). Green leaves of plants thus absorb a substantial fraction of green light as well, also because it penetrates deeper into mesophyll as compared to blue and red lights (Brodersen and Vogelmann 2010). As a result, green light considerably enhances the efficiency of photosynthetic CO_2_ assimilation on the entire leaf level (particularly under moderate and strong white light) (Terashima et al. 2009).

In the natural environment fully monochromatic light does not occur. However, distinct changes in spectral quality of light can be affected by penetration through different canopies (Urban et al. 2007; Navrátil et al. 2007), clouds or by season of the year (Opálková et al. 2018). Nevertheless, the controlled growing systems with artificial lighting are increasingly used for enhancing production of crop plants, as well as accumulation of bioactive compounds (Darko et al. 2014; Hamdani et al. 2019; Hasan et al. 2017). With the development of light-emitting diodes (LEDs), providing single colors in the range from ultraviolet to infrared, LEDs have become an innovative light source for such systems (Ouzounis et al. 2015; Bourget 2008) and simplified and boosted research on the effects of monochromatic light treatment (Landi et al. 2020). In indoor agriculture the spectral characteristics of LED light sources are crucial for plant growth and quality via the regulation of photosynthesis, photomorphogenesis and secondary metabolism. Thus, effects of single colors, including green, become more physiologically relevant (He et al. 2019).

Long term exposure (acclimation or growth) of plants to monochromatic green light adversely affects plant growth, as documented by the impairment of several morphological and anatomical parameters in many plant species [for a review, see Landi et al. (2020)]. Some effects of green light are dependent on known sensory systems of photoreceptors (Sellaro et al. 2010), others are independent on photoreceptors, but induced by the shade avoidance symptoms, as revealed by studies using cryptochrome and phytochrome mutants of *Arabidopsis* (Zhang et al. 2011) [for a review, see Battle et al. (2020)]. The impairment of plant growth under green light can partly be attributed to negative impacts on the photosynthetic apparatus. From several studies it is possible to conclude that plants grown under monochromatic green light have lower photosystem II (PSII) activity (*F*_V_/*F*_M_), electron transport rate and CO_2_ assimilation efficiency (Muneer et al. 2014; Su et al. 2014). Further, lower chlorophyll (Chl) content and decreased Chl *a*/*b* ratio was observed for plants grown under green LEDs as compared to plants grown under white, blue or red light (Mizuno et al. 2011; Su et al. 2014), although the impact of green light on Chl content can vary greatly [for a review, see Landi et al. (2020)], probably due to the different plant species, intensity of the growth light, spectral characteristics of the LEDs used or other experimental conditions.

Recently, we have demonstrated that leaves of barley and other plant species grown under monochromatic green light (GL) accumulated a large pool of geranylgeranyl-Chl *a*, Chl intermediates with incomplete hydrogenation of their phytyl chains (Materová et al. 2017). The effect of GL on this step is surprising because in the Chl *a* synthesis pathway the only light-dependent process is the conversion of protochlorophyllide to chlorophyllide (Reinbothe et al. 2010; Schoefs 1999; Iñigo et al. 2012). After this step, two pathways leading to final production of Chl *a* have been described (Shpilyov et al. 2005). Mostly, binding of geranylgeranyl diphosphate (GGPP) to chlorophyllide occurs, followed by hydrogenation of the phytyl chain (Bollivar 2006). Alternatively, GGPP is reduced to phytyl diphosphate (phytyl-PP), followed by binding of phytyl-PP to chlorophyllide (Shpilyov et al. 2005). In both pathways, geranylgeranyl reductase (GGR), which is encoded by *chlp* gene, has a crucial role (Giannino et al. 2004; Park et al. 2010; Wang et al. 2014), either reducing geranylgeranyl Chl (Chl_GG_) into phytyl Chl (Chl_phy_) via dihydrogeranylgeranyl Chl (Chl_DHGG_) and tetrahydrogeranylgeranyl Chl (Chl_THGG_), or reducing GGPP to phytyl-PP, thus providing phytyl residues, not only for Chl synthesis but tocopherol and phylloquinone as well (Keller et al. 1998; Tanaka et al. 1999). Further, it has been suggested that the light-harvesting-like LIL3 proteins are involved in the formation of phytyl chains by stabilizing GGR (Tanaka et al. 2010; Takahashi et al. 2014) serving as an anchor for GGR to the membrane through LHC motif (Lohscheider et al. 2015; Mork-Jansson et al. 2015; Tanaka et al. 2010). *Arabidopsis* plants have two *lil3* gene copies. Both single *lil3* mutants accumulated a minor fraction of geranylgeranylated Chls without any distinguishable phenotypes from wild type. However, the *lil3* double mutant accumulated the majority of geranylgeranylated Chls and exhibited yellowish green leaves and retarded growth rate (Tanaka et al. 2010).

In GRR-deficient plants and cyanobacterial mutants, the geranylgeranylated Chls, which are accumulated instead of phytylated Chls, are incorporated into photosynthetic PPCs and even mediate light-induced electron transport in the mutants (Tanaka et al. 1999; Shibata et al. 2004a, b; Shpilyov et al. 2005, 2013). As mentioned above, we have shown that also wild-type plants, after several days exposure to monochromatic green light, accumulate geranylgeranylated Chls to a large extent (Materová et al. 2017). Accumulation of geranylgeranylated Chl *a* exerted no significant effect on plant growth at 240 μmol photons m^−2^ s^−1^. Also, the light response curves of CO_2_ assimilation in GL- and WL-grown plants were essentially identical, but only at low and moderate light intensities. In high light (> 200 μmol photons m^−2^ s^−1^), the CO_2_ fixation in GL-grown plants was severely retarded compared to the WL-grown control (Materová et al. 2017), suggesting impaired photosynthetic activities. However, information about the distribution of geranylgeranylated Chls in individual PPCs and consequences of these Chls on the assembly and function of PSII complexes is missing.

In this study, we demonstrate that acclimation to green light leads to a predominant accumulation of geranylgeranylated Chls *a* and *b* in *Arabidopsis* leaves. Further, we show that geranylgeranylated Chls are present in all major PPCs and supercomplexes, although their distribution among PPCs is not uniform. Accumulation of geranylgeranylated Chls in *Arabidopsis* leaves acclimated to green light brought about impaired assembly of PSII and PSI supercomplexes and their ordered macro-arrays in the thylakoid membranes, evidently due to lower temperature stability of PPCs, especially that of LHCII trimers. Based on these findings and previous experiments with monochromatic green light (Mizuno et al. 2011; Muneer et al. 2014; Su et al. 2014; Materová et al. 2017) role of geranylgeranylated Chls in impairment of photosynthetic performance of plants acclimated at green light is suggested.

## Materials and methods

### Plant material and growth conditions

*Arabidopsis* (*Arabidopsis thaliana* L. cv. Columbia) plants were grown from seeds under controlled environmental conditions inside a HGC 1014 growth chambers (Weiss, Germany) equipped with white light halogen lamps at a photosynthetic photon flux density (PPFD) of 100 µmol photons m^−2^ s^−1^ in a 8/16 h light/dark cycle and temperature 20 °C. 8 weeks after germination, parts of seedlings were transferred to a FytoScope FS130 growth chamber (Photon Systems Instruments, Drasov, Czech Republic) under green light emitted by LEDs (full width at half maximum 35 nm, with maximum at 535 nm) for 2 weeks while all other controlled parameters remained unchanged, whereas control plants continued to grow under the original white light. Newly formed leaves (formed during the last 2 weeks of cultivation), after at least 2 h-dark adaptation, were used for measurements on the intact leaves and isolation of thylakoid membranes. The acclimation experiment was performed in 4 independent replicates.

### HPLC–DAD estimation of photosynthetic pigment concentration

An extract of photosynthetic pigments was prepared in 100% acetone with the addition of a small amount of MgCO_3_. The extract was centrifuged for 3 min at 3468 × *g* (EBA 20, Hettich, Germany) and diluted to a final concentration of 80% acetone. The supernatant was then filtered through a 0.22 µm PTFE filter (Whatman, UK) into vials.

The photosynthetic pigment extracts were analyzed using an Agilent 1200 HPLC–DAD system (Agilent, USA) equipped with a LiChroCART RP-18 (250 × 4 mm, 5 µm) chromatographic column (Merck, Germany). The separation was performed using two mobile phases (MPs): MP A (acetonitrile/methanol/tris, 241/30/1, v/v/v) and MP B (methanol/n-hexane, 4/1, v/v) with chromatographic column at 22 °C. For the detection of individual pigments, 440 nm light was used. To estimate the relative photosynthetic pigment quantities in *A. thaliana* plants, conversion factors published by Färber and Jahns (1998) were used. For more details see Materová et al. (2017).

### Identification of chlorophyll species with different degrees of side chain saturation

Analysis of chlorophylls (Chls) was performed on UltiMate 3000 UHPLC system (Thermo Fisher Scientific, USA) equipped with diode array detector and tandem Q-TOF mass spectrometer (micrOTOF-QII, Bruker Daltonics, Germany). Hypersil Gold RP-C18 chromatographic column (50 mm × 2.1 mm, 1.9 µm particle size, 175 Å pore size, Thermo Fisher Scientific, USA) was used for the separation of individual photosynthetic pigments from leaf extracts (see above). The gradient of two mobile phases was used for the analysis: mobile phase A consisted of 20% acetonitrile and mobile phase B of 100% acetonitrile (the gradient is described in detail in Table 1). The flow of mobile phase was set to 0.3 ml min^−1^ and the column compartment temperature was kept at 22 °C during the whole separation process. The 10 µl injection of the leaf extract was used for the analysis. The photosynthetic pigments were detected at 440 nm, and the absorption spectra were collected in the range of 190–750 nm. The mass spectrometry (and MS/MS) analysis was performed in the positive ion mode (electron spray ionisation—ESI; end plate offset: − 500 V, capillary voltage: 4500 V, nebulizer gas pressure: 350 kPa, dry gas flow: 8 l min^−1^, dry temperature: 200 °C; inert gas: N_2_). The full-scan mass spectra were collected in the range of 50–1500 m/z. Collision-induced dissociation—CID (at 35 eV) was used for the fragmentation of Chl [M + H]^+^ quasi-molecular ions and subsequent structural analysis.

**Table 1:** The gradient of mobile phases (M.F.) used for the chromatographic separation of photosynthetic pigments (including Chl species with different degrees of side chain saturation) present in *Arabidopsis* leaf extracts

Time of analysis [min]	0	18	25	27	34	+ 3 min post-time
M.F. A: 20% acetonitrile	50	0	0	50	50	
M.F. B: 100% acetonitrile	50	100	100	50	50	

### Chlorophyll a fluorescence measurements

Fast Chl *a* fluorescence induction (OJIP) transients were measured at room temperature on the adaxial side of freshly detached leaves from dark-adapted plants. OJIP transients were measured using a portable fluorometer (FluorPen FP 100, Photon Systems Instruments) in the middle of leaf segments for 2 s. For excitation, blue light emitting diodes provided 3000 μmol photons m^−2^ s^−1^.

The slow kinetics of Chl *a* fluorescence induction and relaxation was measured on dark-adapted leaves using PAM 101/103 fluorometer equipped with the emitter-detector unit ED-101BL employing a blue LED as a source of excitation light (H. Walz, Effeltrich, Germany). Chl fluorescence was detected above 660 nm. First, *F*_V_/*F*_M_ = (*F*_M_ − *F*_0_)/*F*_M_ parameter, characteristic for PSII activity, was determined by means of 800 ms-saturating light pulse with intensity of approx. 5000 µmol photons m^−2^ s^−1^. F_0_ and F_M_ are minimal and maximal fluorescence levels in dark-adapted state, respectively. Then, leaves were exposed to actinic white light with an intensity of 1200 µmol photons m^−2^ s^−1^ for 10 min together with saturating pulses in 1-min intervals. To follow the 5 min-dark relaxation phase, the actinic light was switched off and saturating pulses were applied in 1-min intervals. The following fluorescence parameters were determined: Stern–Volmer non-photochemical quenching of minimal fluorescence, SV_0_ = *F*_0_/*F*_0_′ − 1 (Gilmore and Yamamoto 1991) and relative quenching coefficient for non-photochemical quenching, *q*_(N)rel_ = (*F*_M_ − *F*_M_’)/(*F*_M_/*F*_0_′) (Buschmann 1995; Gáspár et al. 2006). *F*_0_′ and *F*_M_′ are minimal and maximal fluorescence, respectively, during illumination or dark relaxation.

### Isolation of thylakoid membranes and clear-native polyacrylamide gel electrophoresis (CN-PAGE)

Isolation of thylakoid membranes was performed as previously described (Karlický et al. 2016). Before CN-PAGE analysis the thylakoid membranes were solubilized for 5 min with 10% (w/v) detergent n-dodecyl-α-D-maltoside (α-DM) to yield a ratio of detergent to total Chls of 25:1 (w/w). Then high-speed centrifugation (21,000 × *g* for 10 min) was used to remove unsolubilized thylakoid membranes. The supernatant containing thylakoid membranes was immediately loaded onto polyacrylamide gel (15 μg of total Chls to well). Separation of PPCs by CN-PAGE was carried out according to (Karlický et al. 2016) with slight modification—Bis–Tris system of cathode and anode buffers (pH 7.0) were used with addition of 0.02% w/v α-DM (instead of β-DM) and anionic detergent sodium deoxycholate (0.05% w/v) to cathode buffer.

Images of gels containing separated PPCs were captured by ChemiDoc MP gel imager (Bio-Rad Laboratories, Hercules, CA, USA) in transmitting white light or Chl *a* fluorescence excited by blue light with CCD detection. Relative amounts of individual PPCs were compared using one-dimensional densitograms calculated from the green gel images in the Matlab software procedure according to Ilík et al. (2002) or Karlický et al. (2016). Briefly, the 16-bit transmission images were corrected for the background noise, by applying a threshold value, and were transformed into optical density images. Relative contents of the proteins were determined using a half-automated quantitative analysis of different bands via calculating the integrated optical density of the selected region of interest.

To release individual PPCs from gel, the corresponding bands were cut out of the gel, sliced by scalpel and immersed in storage medium (400 mM sucrose, 15 mM NaCl, 5 mM MgCl_2_, 50 mM HEPES, pH 7.2) supplemented with 0.03% or 0.008% w/v α-DM for LHCII and other PPCs, respectively, to overnight spontaneous diffusion (at 4 °C). Eluted PPCs were concentrated by Amicon Ultra 4 Centrifugal filters and used for spectroscopic and pigment analysis (after extraction in 100% acetone, as described above).

### Chlorophyll a fluorescence spectroscopy

Chl *a* fluorescence spectra at 77 K were recorded on thylakoid membranes and intact leaves using a luminescence spectrofluorimeter LS50B (Perkin-Elmer, Beaconsfield, United Kingdom) equipped with the custom-made Dewar-type optical cryostat as previously described (Karlický et al. 2010). Thylakoid membrane samples for fluorescence spectroscopy were prepared from frozen (77 K) suspension that were thawed and diluted in medium containing 50 mM Tricine (pH 7.5), 0.4 M sorbitol, 5 mM KCl and 5 mM MgCl_2_ to a Chl content of 5 μg ml^−1^ to avoid reabsorption. The emission spectra were recorded at the preferential excitation of Chl *b* at 476 nm or Chl *a* at 436 nm, with 5 and 2.5 nm slit widths of excitation and emission monochromators, respectively. The emission spectra were corrected for the spectral sensitivity of the detection system.

For a more detailed analysis, 77 K Chl *a* fluorescence emission spectra of thylakoid membranes were decomposed into its main components according to Andreeva et al. (2003). The experimental emission spectra were fitted with the sum of Gaussian bands (the six main bands and three small vibrational subbands) using the least square method. The main emission bands correspond to the emitting PPC in thylakoid membranes: free LHCII trimers and monomers emit a peak with maximum at 680 nm (denoted as F680); PSII core antenna proteins CP43 and CP47 at F685 and F695, respectively; aggregated LHCII trimers at F700; core complex of PSI at F720 and LHCI at F735. The area under each emission peak was calculated and used to estimate relative changes.

### Absorbance and circular dichroism spectroscopy

Absorption spectra in the range of 350–750 nm were recorded at room temperature with a Specord 250 spectrophotometer (Analytik Jena, Jena, Germany) in steps of 0.2 nm with a band-pass of 0.5 nm and scanning speed of 1 nm s^−1^ in the cell with optical pathlength of 1 cm. Room temperature CD spectra of thylakoid membranes were recorded in the range of 400–750 nm with a J-815 spectropolarimeter (Jasco, Tokyo, Japan). The spectra of thylakoid membranes were recorded in steps of 0.5 nm with an integration time of 1 s, a band-pass of 2 nm and scanning speed of 100 nm min^−1^ in the cell with optical pathlength of 1 cm. Stacked thylakoid membranes were prepared by resuspension of fresh isolated thylakoid membranes at a Chl content of 20 μg ml^−1^ in medium containing 50 mM Tricine (pH 7.5), 0.4 M sorbitol, 5 mM KCl and 5 mM MgCl_2_. In order to obtain unstacked thylakoids displaying no Ψ-type CD bands, the thylakoid membranes at the same Chl concentration were washed in 50 mM Tricine buffer supplemented with 5 mM MEDTA (pH 7.5) and were sonicated (GM 3100; Bandelin Electronic, Berlin, Germany) on ice for 300 s using 0.5 s duty cycle and output value of 25%. Solubilized thylakoid membranes were prepared by 5 min solubilization with 10% (w/v) detergent n-dodecyl-β-D-maltoside (β-DM) to yield a ratio of detergent to total Chls of 20:1. High-speed centrifugation (21,000 × *g* for 10 min) was used to remove unsolubilized thylakoid membranes. The supernatant with solubilized thylakoid membranes was diluted with 20 mM Tricine buffer (pH 7.5) to a Chl content of 20 μg ml^−1^ and 0.1% β-DM concentration and used for CD measurement. LHCII trimers were eluted during night at 4 °C, from cut CN-PAGE gel strips, concentrated with 30 kDa cutoff Amicon filters (Millipore) and then diluted in 20 mM Tricine buffer (pH 7.8) with 0.03% α-DM (for solubilized trimers) to a unit absorbance at the red maximum. Intact leaves were supported by a flat cell and CD spectra were measured, with a measuring beam perpendicular to the leaf, with 3 nm band-pass to improve the signal-to-noise ratio. For temperature dependences of CD spectra, between 20 and 70 or 80 °C, the samples were thermostated in Peltier sample holder (Jasco, Tokyo, Japan), with 5 min incubation at each measuring temperature before recording the spectra. CD spectra were normalized to the Chl Q_y_ absorption band. In order to minimize the influences of the overlapping excitonic CD bands, the amplitudes of the (+) 685 nm, (−) 673 nm and (+) 505 nm Ψ-type CD bands were calculated as the difference of the CD signal at 685 to 750 nm, 673 to 620 nm and 505 to 620 nm, respectively.

### Statistical analysis

Mutual comparison of samples from GL- and WL-acclimated plants was carried out using t-test. Statistical analysis of the amounts of geranylgeranylated Chls in pigment-protein complexes (PPCs) relative to that of thylakoid membranes was performed separately for Chl *a* and Chl *b* using Kruskal–Wallis ANOVA followed by Dunn’s post hoc test or ANOVA followed by Tukey’s post hoc. If the *P*-value of a test was lower than 0.05 or even lower than 0.01 or 0.001, the results were marked by *, ** or ***, respectively. Absence of above mentioned symbols or ns indicates non-significant differences among sample sets. All testing was performed using Origin 8.6 (OriginLab, Northampton, USA).

## Results

In order to reliably determine the effect of geranylgeranylated Chls *a* and *b* on the organization and stability of PPCs in the thylakoid membrane, plant material containing a more pronounced amount of these Chls *a* and *b* was required than in our previous study on barley, in which maximally 15% geranylgeranylated Chl *a* and only trace amounts of Chl *b* accumulated in plants grown under green light from the seed for two weeks (Materová et al. 2017). This was achieved by a two-week long acclimation of *Arabidopsis* plants in green light after eight-week precultivation period in white light. Under these conditions, the leaves that were newly developed during green light acclimation, used here for all experiments, contained more than 50% of geranylgeranylated Chls *a* and *b*.

### Identification of chlorophyll species present in plants cultivated under green light conditions

**Fig. 1:**
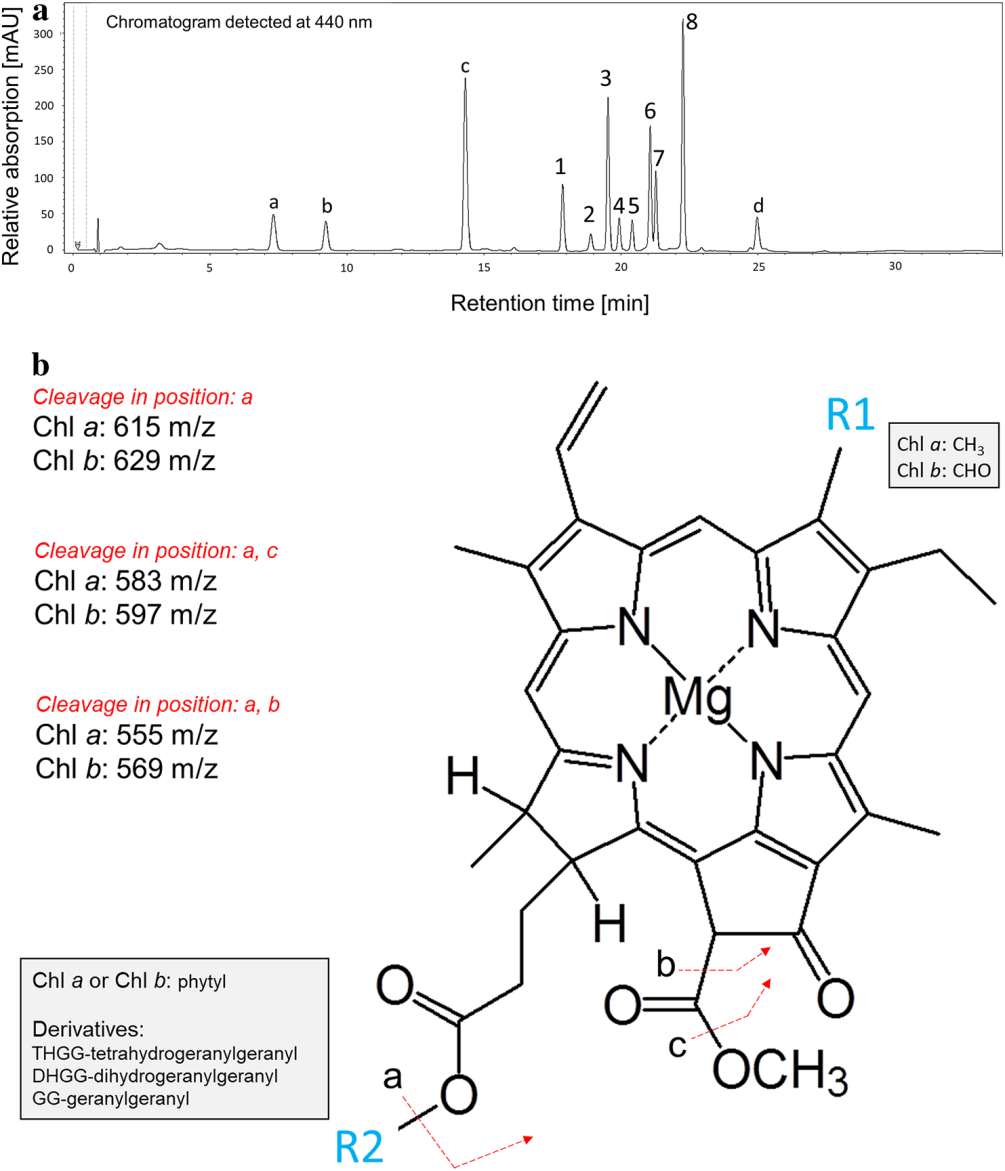
Identification of chlorophyll molecules. **a** HPLC separation of photosynthetic pigments present in leaf extract from *Arabidopsis* leaves developed during the 2-week-long acclimation of plants to green light. The chromatogram was detected at 440 nm. Under the current chromatographic conditions, the retention time of Chl molecules with incomplete hydrogenation of their phytyl chains decreases with the number of double bonds located on the phytyl side chain (Chl_GG_ < Chl_DHGG_ < Chl_THGG_ < Chl). Peak 1—Chl b_GG_, 2—Chl b_DHGG_, 3—Chl a_GG_, 4—Chl b_THGG_, 5—Chl a_DHGG_, 6—Chl b, 7—Chl a_THGG_, 8—Chl a. The additionally detected peaks are attributed to carotenoids (a—neoxanthin, b—violaxanthin, c—lutein + zeaxanthin, and d—β-carotene; identification of these pigments was not in the scope of this study). **b** Proposed molecular cleavages explaining the observed fragment ions after CID of Chl a, Chl b species (based on Chl a fragmentation presented by Wei et al. 2013). Chl a species exhibited fragments 615, 583, and 555 m/z, whereas Chl b species 629, 597, and 569 m/z

Identification of Chl species in the pigment extracts was performed based on their optical properties (UV–VIS absorption spectra), mass spectra (MS) and fragmentation spectra of parent quasi-molecular ions (MS/MS) after the HPLC separation of individual photosynthetic pigments. The Chl species were well separated and eluted within the retention time (Rt) range of 17–23 min (see Fig. 1a; Table 2). Peaks of Chl *a* (Fig. 1a; Peak 8, Rt = 22.3 min) and Chl *b* (Fig. 1a; Peak 6, Rt = 21.1 min) exhibited typical Chl absorption spectra (Soret and Q_y_ bands) with local maxima at 430, 662 nm and 457, 646 nm, respectively. Their identity was further confirmed by the presence of [M + H]^+^ ions 893.54 m/z (Peak 8) and 907.52 m/z (Peak 6) which were previously reported [e.g. Chen et al. (2015)] and are in accordance with Chl *a* and Chl *b* molecular formula. Importantly, the same light absorption maxima (± 1 nm) were observed in case of all other Chl species (see Table 2) suggesting that the structural differences amongst Chls and their respective species are located on the phytyl side chain rather than on the tetrapyrrole structure, which is primarily responsible for their absorption properties in the visible region of the electromagnetic spectrum. MS analysis of the peaks belonging to earlier eluting species of Chl *a* (Fig. 1a; Peaks 3, 5, 7) led to detection of [M + H]^+^ 887.49, 889.50, and 891.54 m/z ions. This pattern indicated increasing number of unsaturated bonds (compared to Chl *a*; 893.54 m/z) in phytyl chain of corresponding Chl *a* species (mass difference corresponding to the loss of 6, 4 and 2 hydrogen atoms, respectively). The same trend was observed for Chl *b* species (Fig. 1a; Peaks 1, 2 and 4), see Table 2. The CID fragmentation of Chl *a* and three other detected Chl *a* species led to the production of the same fragment ion 615.2 m/z (or 629.2 m/z in case of Chl *b* species). Since these ions belong to the tetrapyrrole part (chlorophylide-like part) of the molecule (see Fig. 1b, cleavage in position—a), the observed mass difference of Chls and their respective species (see Table 2) can only be attributed to the different mass of the phytyl part of the molecule (i.e. to R2 residue; see Fig. 1b). In agreement with Mizoguchi et al. (2017), we conclude that the detected Chl species contain tetrahydrogeranylgeranyl (THGG), dihydyrogeranylgeranyl (DHGG) or geranylgeranyl (GG) side chains instead of the phytyl. The other two fragment ions detected after CID of Chl *a* species were m/z 583 and 555 (597 and 569 in case of Chl *b* species). According Wei et al. (2013), these ions originate from further cleavages of the side chain located at heterocycle V (see Fig. 1b, cleavage a, c resp. a, b). The presence of these ions in the fragmentation spectra of Chl species support our conclusion that the structural differences amongst the observed compounds are due to alterations in the R2 residue (Table 1; Fig. 1b).

**Table 2:** Summary of parameters used for the identification of Chl a and Chl b species with altered phytyl chains

Peak*	Compound	Rt [min.]	VIS λ_max_ [nm]	MS m/z	MS/MS (CID 35 eV) m/z
1	Chlorophyll *b*_GG_	17.89	458; 647	901.4722	629.2194; 569.1931; 597.1968; 647.2182
2	Chlorophyll *b*_DHGG_	18.91	458; 647	903.484	629.2194; 597.1968; 569.2083
3	Chlorophyll *a*_GG_	19.56	430; 663	887.4905	615.2443; 555.2230; 583.2145
4	Chlorophyll *b*_THGG_	19.96	457; 647	905.5025	629.2224; 597.1918; 569.2022
5	Chlorophyll *a*_DHGG_	20.43	430; 663	889.5061	615.2443; 555.2247; 583.2162
6	Chlorophyll *b*	21.08	457; 646	907.5207	629.2241; 569.1904; 597.1968
7	Chlorophyll *a*_THGG_	21.29	430; 663	891.5434	615.2427; 583.2060; 555.2333
8	Chlorophyll *a*	22.29	430; 662	893.5428	615.2398; 584.2098; 555.2161

*Peak number corresponds to chromatogram labelling shown in Fig. 1a

### Geranylgeranylated Chl contents of the pigment-protein complexes

**Fig. 2:**
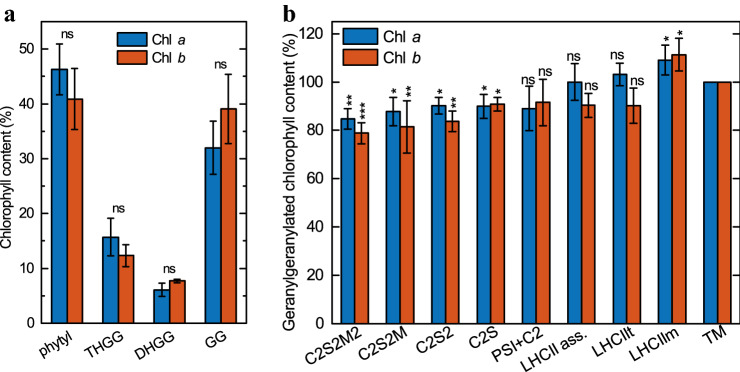
Proportions of phytylated and geranylgeranylated Chls in thylakoid membranes and PPCs. **a** Amounts of phytylated (phytyl), tetrahydrogeranylgeranylated (THGG), dihydrogeranylgeranylated (DHGG) and geranylgeranylated (GG) Chls a and b relative to their total contents in the thylakoid membranes isolated from *Arabidopsis* thaliana leaves developed during GL acclimation of plants. The data are the means of four independent experiments ± SD. Asterisks indicate statistically significant differences (Student’s t-test) between GL and WL (*P < 0.05; **P < 0.01; ***P < 0.001). **b** Amounts of geranylgeranylated Chls a and b (sum of THGG, DHGG and GG Chls) in different PPCs relative to their amounts in the thylakoid membranes from which the separation was performed. In the thylakoid membranes, the geranylgeranylated Chls a and b was present at 53.3 ± 3.8% and 58.6 ± 4.6%, respectively. The data are the means of three independent measurements ± SD. Asterisks indicate statistically significant differences (Kruskal–Wallis ANOVA followed by Dunn’s post hoc test) between the given PPC and the thylakoid membranes from which the separation was performed (*P < 0.05; **P < 0.01; ***P < 0.001)

Acclimation of *Arabidopsis* plants to GL induced a decrease in total Chls and carotenoids (data not shown) in agreement with a previous report (Materová et al. 2017). In the control plants acclimated to WL, the geranylgeranylated Chls *a* and *b* were clearly detectable (almost exclusively as Chl_THGG_), nevertheless their amount was always less than 4% of the total Chls contained in thylakoid membranes (data not shown). In contrast, the leaves grown on plants acclimated to GL contained large amounts of geranylgeranylated Chls *a* and *b* and all intermediates of the three-step reduction of geranylgeranylated Chl to phytylated Chl (Chl_GG_ was the most abundant), and less than 50% of the Chls *a* and *b* contained fully reduced phytyl chains (Fig. 2a).

In order to determine the distribution of geranylgeranylated Chls *a* and *b* among different PPCs, we estimated the pigment contents of PPCs separated by CN-PAGE. As shown in Fig. 2b, the geranylgeranylated Chls *a* and *b* were present, albeit not in equal proportions, in all PPCs and supercomplexes. The distribution of geranylgeranylated Chls *a* and *b* between the individual PPCs indicated increasing proportion of geranylgeranylated Chls with decreasing size of the PPCs (Fig. 2b). The largest investigated PPC, the biggest PSII supercomplex called C2S2M2, contained almost 20% less and the smallest studied PPC, the LHCII monomers, roughly 10% more geranylgeranylated Chls compared to the average value in the thylakoid membrane. The differences in the distribution of geranylgeranylated Chls are statistically significant especially between PSII supercomplexes and LHCII containing zones (Table S1).

In order to assess the effect of acclimation of plants at GL (associated with the accumulation of geranylgeranylated Chls *a* and *b*) on the carotenoid composition in PPCs, composition of all photosynthetic pigments in the PPCs and thylakoid membranes of GL- and WL-acclimated *Arabidopsis* were measured (Fig. S1). On the level of thylakoid membranes, the Chl *a*/*b* and Car/Chl ratios, including the carotenoid composition, changed after the acclimation to GL as compared to WL thylakoid membranes. These changes can be explained by variations in the PPC composition of the thylakoid membranes after acclimation to GL as discussed below. Above all, higher levels of LHCII with a high content of Chl *b*, lutein, neoxanthin and violaxanthin in the GL thylakoid membranes compared to WL, were obtained. This brings about a decrease in β-carotene content, which is not found in LHCII. Composition of photosynthetic pigments in PPCs isolated from control plants (Fig. S1) was in harmony with data obtained from crystal structures and biochemical analyses (Crepin et al. 2016; Caffarri et al. 2014). The pigment composition of PPCs isolated from GL-acclimated plants clearly showed that neither the Chl *a*/*b* nor the amount of total carotenoids and their composition were considerably affected by the presence of geranylgeranylated Chls *a* and *b*. Only PSII supercomplexes from GL-acclimated plants revealed statistically significant increase in violaxanthin content, which was accompanied by a slight, statistically insignificant decrease in lutein content (Fig. S1).

### Clear-native gel electrophoresis of WL- and GL-acclimated thylakoid membranes

**Fig. 3:**
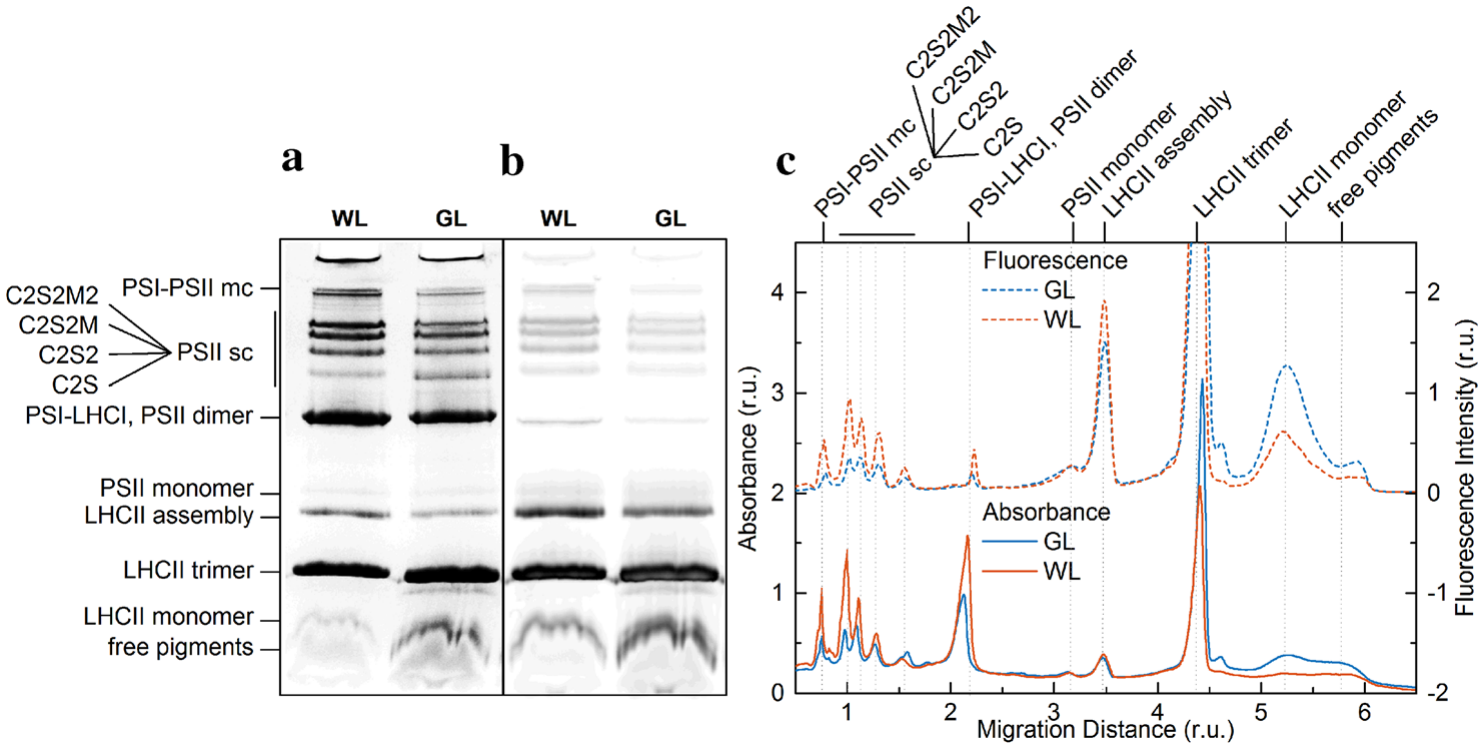
CN-PAGE separation of PPCs of thylakoid membrane obtained from *Arabidopsis* thaliana leaves acclimated to green (GL) and white (WL) light. **a** Gel images were obtained as transmittance of white light or **b** Chl a fluorescence, excited by blue light, using CCD camera ChemiDoc MP (Bio-Rad Laboratories). Typical electrophoretograms from three independent experiments with a very similar result. **c** One-dimensional densitograms calculated from the green gel images shown in panels a and b. (*sc* supercomplex; *mc* megacomplex.)

Changes in PPC composition of the thylakoid membrane caused by acclimation to GL were also observed using CN-PAGE. Electrophoretograms of control plants (Fig. 3a, b, first lane, WL) revealed typical patterns of the protein complexes of thylakoid membranes after solubilization with DM [cf. e.g. (Järvi et al. 2011)]. The relative contents of individual PPCs of thylakoids isolated from GL-acclimated leaves were considerably different from the control, WL-acclimated thylakoids (Fig. 3a, b), as also evident on the one-dimensional densitograms obtained from the green gel images (Fig. 3c). In general, the solubilized thylakoid membranes isolated from leaves developed in GL, when compared to the control, contained increased amounts of the smaller PPCs at the expense of large supercomplexes (Fig. 3). Especially bands corresponding to PSI-PSII megacomplex and PSII supercomplexes were strongly reduced after acclimation to GL. Moreover, the decrease of PSII supercomplexes compared to WL was more pronounced for larger complexes (Fig. 3c), suggesting that their assembly was hampered in the GL-grown leaves. Based on fluorescence data, the reduction of PSII supercomplexes after acclimation to GL was roughly 70% for C2S2M2, 55% for C2S2M, 50% for C2S2 but only 35% for C2S (Fig. 3c). From the transmittance/absorption gel images and densitograms it might seem that the content of the smallest PSII supercomplex (C2S) is higher in GL-acclimated plants as compared to WL control, but there are several indications that this is caused by a contamination with PSI, presence of which in C2S band is negligible in the control plants. Firstly, the migration distance in the gel (and thus on the densitograms) is slightly shifted, which might lead to an overlap of the two zones. Secondly, disappearance of the shift and relative decrease of the signal in comparison to control thylakoid membranes in Chl fluorescence gel images and densitograms indicate the presence of PSI, because the fluorescence quantum yield of PSI at room temperature is much lower than that of PSII. Finally, contamination by PSI was confirmed by low temperature fluorescence emission spectra of C2S band having significant PSI emission at 735 nm for GL plants in comparison with WL plants (Fig. S2). Further, GL thylakoid membranes appeared to contain less PSI-LHCI and PSII-core dimers (as seen from Chl fluorescence gel images and densitograms), about the same amount of PSII-core monomer and slightly less LHCII assembly (LHCII–CP29–CP24). On the other hand, electrophoretic separation of GL thylakoid membranes revealed significantly more LHCII trimers and monomers unbounded in supercomplexes in comparison with WL samples (Fig. 3).

### Chlorophyll a fluorescence transients at room temperature

**Fig. 4:**
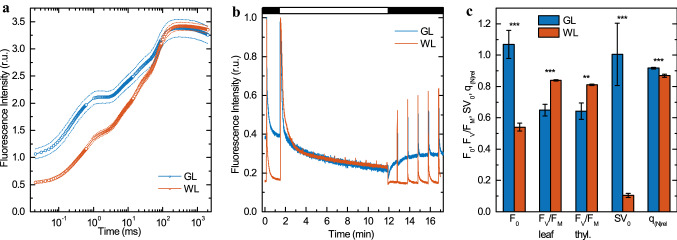
Room temperature Chl a fluorescence analysis of detached *Arabidopsis* leaves acclimated to light intensity of 100 μmol photons m^−2^ s^−1^ of either green (GL) or white (WL) light. **a** Fast Chl a fluorescence induction (OJIP) transient. **b** Representative traces of room temperature Chl a fluorescence analysis for leaves illuminated with 1200 µmol photons m^−2^ s^−1^ of actinic light. **c** Related fluorescence parameters: a minimal fluorescence (*F*_0_) calculated from OJIP transients shown in Panel a, the fluorescence parameter *F*_V_/*F*_M_ = (*F*_M_ − *F*_0_)/*F*_M_ characteristic of PSII activity, measured on intact leaves (calculated from the transients shown in Panel c) and on isolated, fully functional thylakoid membranes), steady state level of non-photochemical quenching of minimal fluorescence (SV_0_ = *F*_0_/*F*_0_′ − 1) and relative quenching coefficient for non-photochemical quenching (q_(N)rel_ = (*F*_M_ − *F*_M_′)/(*F*_M_/*F*_0_′)) calculated from the transients shown in Panel c. Results are averages ± SD obtained from measurements on 5–12 leaves and on four independently isolated thylakoid membranes. Asterisks indicate statistically significant difference (Student’s *t*-test) between GL and WL (*P < 0.05; **P < 0.01; ***P < 0.001)

In order to determine the performance of photosynthesis, especially that of PSII, several types of room temperature kinetic Chl *a* fluorescence measurements were performed. The OJIP transients in the GL-acclimated *Arabidopsis* leaves shows several differences such as the higher initial O-J phase, but by far the most prominent feature is higher F_0_, which may originate from energetically uncoupled light-harvesting antenna complexes (Fig. 4a).

Increase in F_0_ value were accompanied by significant reduction of the *F*_V_/*F*_M_ parameter (Fig. 4b, c), characteristic of the photochemical activity of PSII, in leaves acclimated to GL (0.649 ± 0.037) in comparison with the WL-grown leaves (0.839 ± 0.004) (Fig. 4c). It is worth noting, that the same *F*_V_/*F*_M_ values were estimated for isolated thylakoid membranes showing their full functionality, which is important for other measurements on thylakoid membranes. For the thylakoid membranes only a negligible decrease of *F*_V_/*F*_M_ parameter occurred during their preparation in comparison with intact leaves (Fig. 4c).

In quenching analysis, the fluorescence signal of GL leaves dropped below F_0_ after turning on the actinic light (Fig. 4b), which reflects non-photochemical quenching of minimal fluorescence, SV_0_ (Fig. 4c). The most commonly used quenching parameter, non-photochemical quenching (NPQ, F_M_/F_M_′ − 1), is unreliable for comparison of GL- and WL-acclimated leaves due to twofold difference in F_0_ and the fact that NPQ refers to absolute values of fluorescence yields (Gáspár et al. 2006). Therefore, relative quenching coefficient for non-photochemical quenching (*q*_(N)rel_) were calculated (Fig. 4c). GL-acclimated leaves had increased *q*_(N)rel_ as compared to WL (Fig. 4c).

### Fluorescence emission and excitation spectra at 77 K

**Fig. 5:**
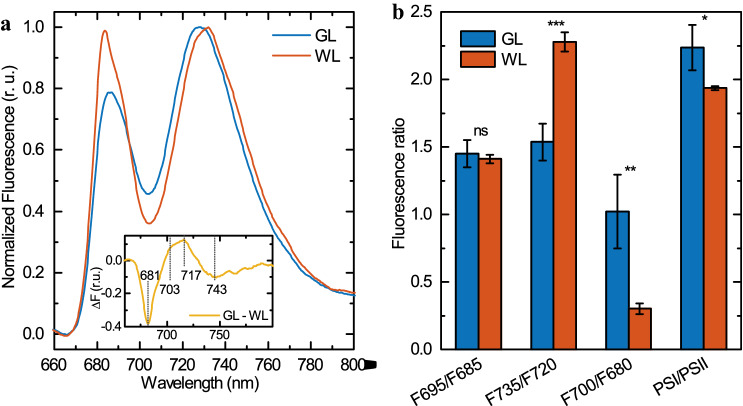
Chl a fluorescence spectra at 77 K. **a** 77 K Chl a fluorescence emission spectra of *Arabidopsis* thylakoids isolated from dark-adapted leaves acclimated to green (GL) and white (WL) light, normalized to PSI emission; and WL-GL difference spectra (inset). The emission spectra were excited at 476 nm (preferential excitation of Chl b). Average spectra from four independent experiments. **b** The ratios of areas under Gaussian bands of the selected PPCs (see Fig. S5 for details). Mean values and standard errors from 4 Gaussian decompositions on different batches. Asterisks indicate statistically significant difference (Student’s *t*-test) between GL and WL (*P < 0.05; **P < 0.01; ***P < 0.001)

The 77 K steady-state emission spectrum of thylakoid membranes isolated from control *Arabidopsis* plants (Fig. 5a) revealed the typical features of the higher plants thylakoids, characterized by three clearly recognizable bands, two of which at wavelengths of 685 and 695 nm, are attributed to PSII, and the third band at 735 nm to PSI (Van Grondelle et al. 1994). The low-temperature emission spectrum of GL-acclimated thylakoid membranes was significantly different mainly due to the different shape of PSII emission, the shift of PSI emission maximum by 4 nm to shorter wavelengths and more pronounced fluorescence at 700 nm in comparison with control thylakoid membranes. These differences are better expressed in the differential (GL minus WL) spectrum (Fig. 5a, inset) documenting a lower intensity of fluorescence at 681 nm and higher intensity of fluorescence at 703 nm and 717 nm for GL thylakoids. These specific changes upon GL acclimation are evident on the level of the intact leaf as well (Fig. S3), although the fluorescence spectrum on intact plant tissue is significantly affected by reabsorption and other optical properties of the leaf (Buschmann 2007). Nevertheless, the 77 K fluorescence emission spectra measured on intact leaves document that specific changes upon GL acclimation in the 77 K emission spectra are not caused by isolation of thylakoid membranes. In addition, low-temperature emission spectrum of thylakoid membranes had very similar features in preferential excitation of Chl *b* (476 nm; Fig. 5a) and Chl *a* (436 nm; Fig. S4) indicating no emission from disconnected Chls.

To get more detailed information about the contributions to total fluorescence emission from individual PPCs we performed Gaussian deconvolution analysis of the emission spectra (Fig. S5). This analysis clearly revealed that all differences in the emission spectrum between GL- and WL-acclimated plants can be explained by changes in the intensity of the individual emission bands. Fig. S5c displays contributions to total fluorescence emitted by the main PPC of PSII and PSI, confirming that the major changes take place in the F680, F700 and F720 bands. GL-acclimated plants revealed substantially (by about a factor of two) reduced emission from LHCII (F680), and almost twice as intense emission from red LHCII (F700), as the WL-thylakoids; GL-thylakoids also displayed an approximately 40% increase in PSI core emission in comparison with WL. These pieces of information were clearly supported by the data from fluorescence ratios: the emission ratio F700/F680 increased roughly 3.5 times for GL-acclimated plants, F735/F720 dropped by a third, while the ratio of total PSI and PSII emissions grew only by 15% and the emission ratio of the PSII core antennas F695/F685 remained unchanged (Fig. 5b).

### Absorption and circular dichroism spectra

**Fig. 6:**
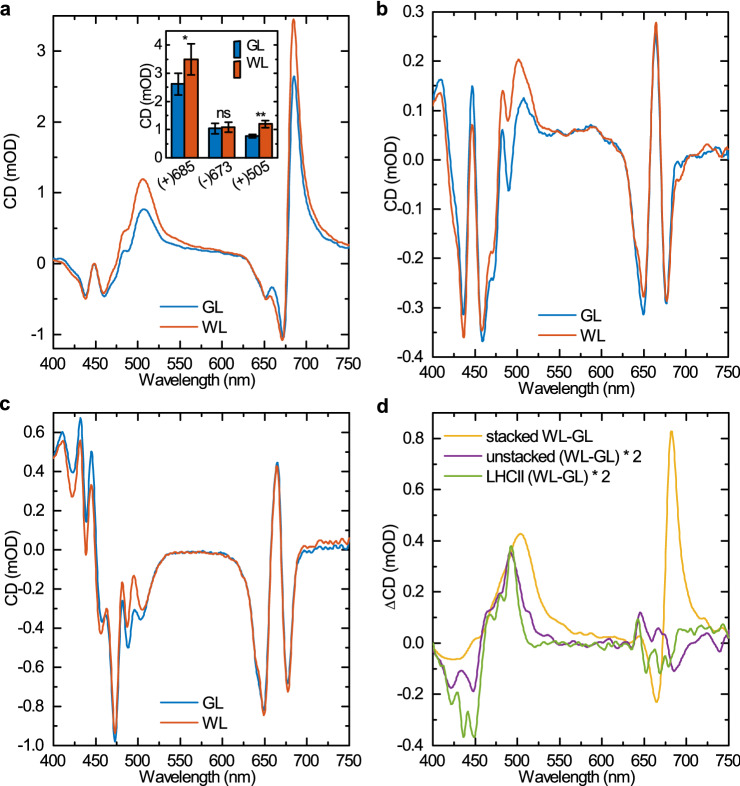
Circular dichroism spectra. CD spectra of thylakoids isolated from dark-adapted WL- and GL-acclimated *Arabidopsis* leaves in stacked (**a**) and unstacked (**b**) states. **c** CD spectra of isolated LHCII trimers isolated from GL and WL thylakoids. **d** CD difference spectra (WL minus GL) of stacked and unstacked thylakoid membranes and isolated LHCII trimers displayed in panels a–c. The spectra are normalized to the Q_y_ absorbance maxima. Average spectra from four independent experiments. Inset in a: amplitudes of the three Ψ-type CD bands of stacked thylakoid membranes; means ± SD (*n* = 4); asterisks indicate statistically significant differences (Student’s *t*-test) between GL and WL (*P < 0.05; **P < 0.01; ***P < 0.001)

Circular dichroism (CD) spectroscopy provides information on both the molecular organization of PPCs via the short-range excitonic interactions (excitonic bands) and on the chiral macro-organization of LHCII trimers and LHCII–PSII supercomplexes, which give rise to intense Ψ-type CD bands (Ψ, polymer or salt-induced) (Garab and van Amerongen 2009; Garab 2016, 2014; Lambrev and Akhtar 2019). The absorption spectra of the control and GL-acclimated samples showed almost no differences under both stacking and unstacking conditions (Fig. S6). As shown in Fig. 6a, the CD spectra of stacked thylakoid membranes from WL-grown and GL-developed leaves revealed similar character as described previously (Krumova et al. 2010; Tóth et al. 2016). The three main CD bands at around (+) 685 nm, (−) 673 nm and (+) 505 nm, which are of Ψ-type origin (Barzda et al. 1994; Dobrikova et al. 2003), and four less intense CD bands of excitonic origin at (−) 438 nm, (+) 448 nm, (−) 459 nm and (−) 650 nm (Georgakopoulou et al. 2007; Garab and van Amerongen 2009) were present in both GL and WL thylakoid membranes. However, the amplitudes of the main Ψ-type CD bands were reduced after acclimation to GL (that of (+) 685 nm by 24% and that of (+) 505 nm by 36%) exhibiting statistically significant differences between from WL and GL samples (Fig. 6a). In agreement with earlier observations, demonstrating that CD spectroscopy can be used in vivo, on whole leaves (Kovács et al. 2006; Tóth et al. 2016), the CD spectra measured for detached leaves form GL- and WL-acclimated plants revealed very similar differences (Fig. S7), confirming that the macro-organization was not significantly affected during isolation of thylakoid membranes.

Because differences between plants acclimated to GL and WL in the excitonic CD bands (dominating in spectral regions of 430–480 nm and 640–660 nm) are difficult to distinguish due to the overlap with more intensive Ψ-type CD bands in the CD spectra of stacked thylakoid membranes, we also recorded the spectra on unstacked thylakoid membranes (Fig. 6b). CD signals under unstacked conditions originate from short-range interactions inside individual PPCs (Garab and van Amerongen 2009), predominantly from excitonic interactions inside LHCII as the most abundant PPC (Georgakopoulou et al. 2007; Lambrev et al. 2007). A comparison of CD spectra of unstacked thylakoid membranes revealed that thylakoids from plants acclimated to GL exhibit significantly different excitonic signals, at least in the blue spectral region, compared to control plants (Fig. 6b). These changes might be accounted for by the altered protein composition (i.e. lower content of core complexes and higher content of LHCII in GL compared to WL thylakoids), but contribution of changes in the LHCII oligomerization state (i.e. LHCII trimer/monomer ratio) or molecular architecture of LHCII compared to the untreated control cannot be ruled out. To clarify the origin of changes in excitonic signals observed in thylakoids from plants acclimated to GL, we measured CD spectra of isolated LHCII trimers as well (Fig. 6c). The GL-WL difference spectra clearly show that at least part of changes in excitonic signals caused by GL acclimation originates from LHCII (Fig. 6c). Figure 6d demonstrates that the most significant difference in excitonic interaction at 492 nm is present at the same magnitude in both the unstacked thylakoids and the isolated LHCII trimers.

### Thermal stability of the thylakoid membrane

**Fig. 7:**
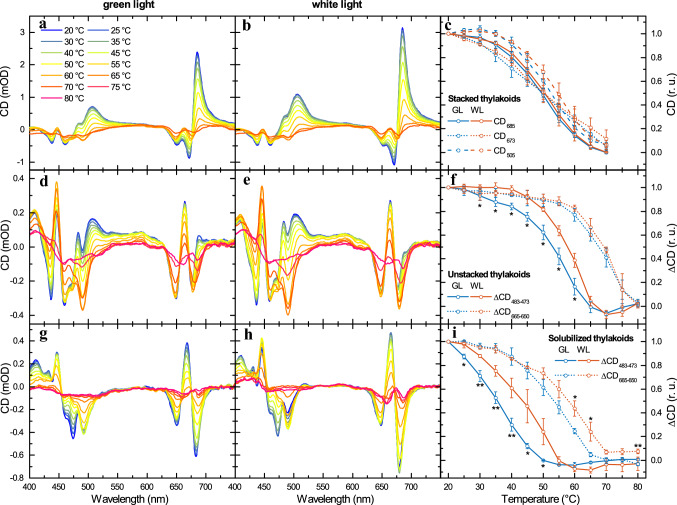
Temperature dependence of the CD spectra of thylakoid membranes and LHCII trimers. Typical CD spectra of stacked (**a**, **b**), unstacked (**d**, **e**) and n-dodecyl-β-maltoside solubilized (**g**, **h**) thylakoid membranes isolated from GL-acclimated (**a**, **d**, **g**) and control (**b**, **e**, **h**) *Arabidopsis* leaves, measured at the indicated temperatures. Temperature dependences of the intensity of the three Ψ-type CD bands (**c**) and of the amplitude differences of the excitonic bands at around (+) 483 nm and (−) 473 nm and (+) 665 nm and (−) 650 nm (**f**, **i**) of samples isolated from GL-acclimated and control (WL) *Arabidopsis* plants. The data points in (**c**, **f** and **i**) are normalized to the respective values at 20 °C. Vertical bars indicate standard errors from four (stacked and unstacked thylakoid membranes) and three (solubilized thylakoid membranes) independent measurements. Asterisks indicate statistically significant differences (Student’s *t*-test) between GL and WL (*P < 0.05; **P < 0.01; ***P < 0.001)

As suggested above, the diminished amounts of PSII-LHCII supercomplexes, together with the decreased size of chiral macro-arrays of PSII-LHCII supercomplexes, in the GL thylakoid membranes, compared to WL, might be connected with lowered stability of PPCs that bind Chls with incompletely reduced phytyl chains. To evaluate the thermal stability of the thylakoid membrane assembly containing PPCs with geranylgeranylated Chls, thylakoid membranes were subjected to gradual heating and CD spectra were recorded at every 5 °C (Fig. 7). Temperature dependences of CD spectra on stacked thylakoid membranes from plants acclimated to GL did not exhibit significant changes in the temperature sensitivity of the Ψ-type CD bands, originating from long-range chiral order of chromophores, compared to WL acclimation (Fig. 7a, b). Differences in the thermal stability of chiral macro-arrays of LHCII trimers and LHCII–PSII supercomplexes can be more clearly seen on the plot of the temperature dependence of the three Ψ-type CD bands (Fig. 7c), showing that the transition temperatures in GL membranes were by no more than 2 °C lower than in the WL membranes. (The transition temperatures for (+) 685 nm, (−) 673 nm and (+) 505 nm Ψ-type CD bands were respectively 50, 49 and 52 °C for GL and 51, 48 and 54 °C for WL.) The higher susceptibility of the (−) 673 nm Ψ-type band to gradual heating compared to the (+) 685 nm and particularly (+) 505 nm bands, is in agreement with the data reported by Cseh et al. (2000) and Kotakis et al. (2018).

In contrast to stacked thylakoid membranes, unstacked GL-acclimated thylakoid membranes exhibited more pronounced diminution of the excitonic bands at elevated temperatures than the WL membranes (Fig. 7d, e). Again, this heat-induced reduction of CD signal can be more clearly seen on the plot of the temperature dependence of the specific CD band pairs (Fig. 7f). The CD band pair of (+) 483 nm/(−) 473 nm, which is specific for LHCII trimers, and thus the disappearance of this band pair indicates the monomerization of the LHCII trimers (Garab et al. 2002; Yang et al. 2006). It can be clearly seen in Fig. 7f that the transition temperature of the monomerization of LHCII trimers in the GL-acclimated sample was lowered by about 5 °C (54 °C) as compared to the control (59 °C), and also to those determined earlier on different plant species (Dobrikova et al. 2003; Krumova et al. 2010; Várkonyi et al. 2009; Petrova et al. 2018). CD bands in red region at (−) 650 nm, (+) 665 nm and (−) 680 nm are present in the CD spectra of both trimeric LHCII and monomeric LHCII (Yang et al. 2006). Therefore, the temperature dependence of the (+) 665 nm/(−) 650 nm band pair indicates disintegration of LHCII monomers. It can be clearly seen in Fig. 7f that the transition temperature of the LHCII monomers disintegration in the GL-acclimated membranes in GL membranes does not differ by more than 2 °C in comparison with WL (transition temperatures are 67 °C for GL and 69 °C for WL).

The thermally induced monomerization of LHCII trimers was also monitored on thylakoid membranes solubilized with n-dodecyl-β-maltoside, by using CD spectroscopy (Fig. 7g, h). Although the temperature stability of LHCII was generally lower in these samples than in the membrane environment, the temperature dependences of the CD band pair of (+) 483 nm/(−) 473 nm confirmed significant difference between GL- and WL-acclimated plants: the trimer-to-monomer transition temperatures were 36 °C for GL and 44 °C for WL (Fig. 7i). Further, the temperature dependence of the diminishment of the CD band pair (+) 665 nm/(−) 650 nm, specific for the disassembly of LHCII monomers, also revealed a lowered thermal stability of GL monomers (54 °C) compared to WL (58 °C) (Fig. 7i).

**Fig. 8:**
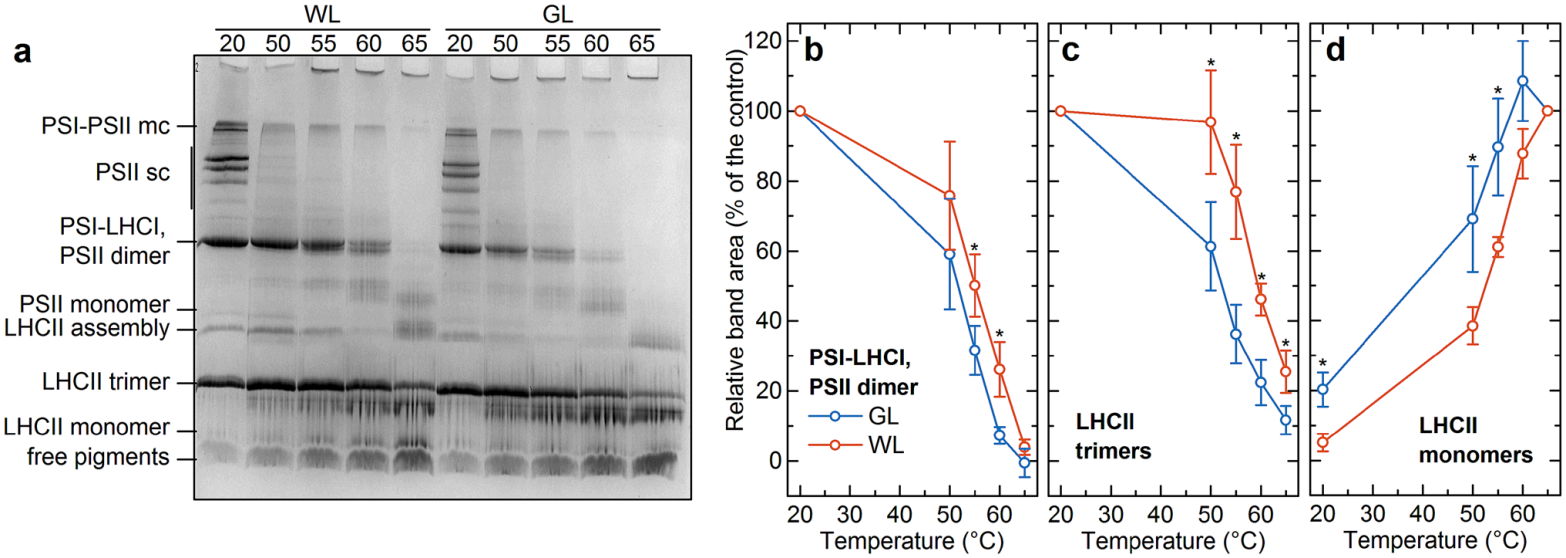
Native green gel analysis of heat-treated thylakoid membranes. **a** Typical CN-PAGE separation of PPCs of heat-treated thylakoid membranes isolated from control (WL) and GL-acclimated *Arabidopsis* plants at different temperatures indicated in the legend. The samples were heat treated for 10 min before loading on the gel. Temperature dependences of the stability of PSI-LHCI supercomplex, co-migrating PSII dimer (**b**) and LHCII trimer (**c**), and the amount of LHCII monomers (**d**) determined from the green gel density profiles of heat-treated thylakoid membranes isolated from GL- (blue) and WL-acclimated (red) leaves. Mean values (points) and standard deviations (error bars) from three independent experiments (*n* = 3). Asterisks indicate statistically significant differences (Student’s *t*-test) between GL and WL (*P < 0.05; **P < 0.01; ***P < 0.001). (*sc* supercomplex; *mc* megacomplex)

In order to confirm the lower thermal stability of LHCII trimers containing geranylgeranylated Chls and to monitor the thermal behavior of the different photosynthetic complexes, we performed green gel electrophoresis on WL and GL thylakoid membranes which were heat treated at different temperatures (Fig. 8a), which revealed prominent differences in the bands assigned to PSI-LHCI, PSII-dimer and those of LHCII. Densitometry analyses of the corresponding bands have shown that both the PSI supercomplexes (combined with PSII dimers) and the LHCII trimers exhibited lower thermal stabilities in GL thylakoid membranes compared to WL: for PSI 50% diminishments were obtained at 52 °C (GL) and 55 °C (WL) (Fig. 8b); for LHCII trimers, these values were found at 52.5 °C (GL) and 59.5 °C (WL) (Fig. 8c). Parallel with the gradual destabilization of LHCII trimers the content of LHCII monomers gradually increased (Fig. 8d); thus, confirming the facilitated monomerization of LHCII in GL membranes, in comparison with WL-thylakoids (Fig. 2d).

## Discussion

### Accumulation of geranylgeranylated chlorophylls impairs photosynthetic performance

Our results clearly show that Chl conjugated with unsaturated geranylgeraniol side chains are contained by all PPCs (Fig. 2b). However, the contribution of Chls with unsaturated phytyl chains relative to the total bound chlorophylls were higher in LHCII than in PSII-LHCII supercomplexes (Fig. 2b, Table S1). To the best of our knowledge, this is the first detailed observation of geranylgeranylated Chls distribution in individual PPCs, although it was shown earlier that PSI accumulate less geranylgeranylated Chls than LHCII trimers (Grasses et al. 2001). Further, it is important to note that the Chl *a*/*b* ratio and relative amount of carotenoids and their composition in individual PPCs appeared to be unaltered after replacement of phytylated Chls by geranylgeranylated Chls (Fig. S1). On the other hand, the PPC composition in GL-acclimated *Arabidopsis* (Fig. 3) and the photosynthetic pigment composition of isolated thylakoid membranes (Fig. S1; last columns), were both changed in a way resembling to lincomycin-treated, LHCII-enriched membranes (Gáspár et al. 2006). Interestingly, similar changes in PPC composition in thylakoid membranes were observed in several plant and cyanobacterial mutants accumulating geranylgeranylated Chls (Shpilyov et al. 2005, 2013; Hey et al. 2017). Shpilyov et al. (2005) demonstrated that when PSI and PSII are assembled with geranylgeranyl Chls, they become unstable and tend to degrade spontaneously during strong illumination. The authors hypothesized that geranylgeranyl residue is more rigid than the phytyl residue due to three additional double bonds and this increased rigidity probably perturbs the association of geranylgeranylated Chls with apoproteins, possibly also disturbing the interaction of protein subunits with each other. Therefore, it seems likely that the changed thylakoid PPC composition in GL-acclimated *Arabidopsis* occurs due to unstable photosystems lacking phytylated Chls rather than as direct acclimation effect of GL. This is in line with the observation that PSII-LHCII supercomplexes have a smaller ratio of geranylgeranylated/phytylated Chls as a result of preferential disintegration of unstable photosystems with geranylgeranylated Chls.

The GL-acclimated *Arabidopsis* leaves had pronouncedly lower PSII activity, as indicated by lower values of the *F*_V_/*F*_M_ parameter compared to the WL control leaves (Fig. 4c). The decrease of this parameter is mainly due to an increase in *F*_0_ (Fig. 4a,c) corresponding to Chls functionally uncoupled to the RCs (Belgio et al. 2012; Gáspár et al. 2006; Ware et al. 2015), which can be attributed to the accumulation of LHCII trimers and monomers in the membrane that are not associated with the super and megacomplexes (Fig. 3b). The twofold increased value of *F*_0_ of GL-acclimated leaves thus appears to be associated with poorly organized PSII supercomplexes and the presence of ‘free’ LHCIIs in GL-acclimated thylakoids (Fig. 3).

Further, GL-acclimated *Arabidopsis* plants revealed a high-Chl fluorescence phenotype (Fig. 4b) and decreased PSII activity (*F*_V_/*F*_M_), whereas *q*_(N)rel_ increase slightly and SV_0_ showed a much larger magnitude than in WL plants (Fig. 4b,c). In addition, GL-acclimated plants revealed considerably limited rapid phase of NPQ relaxation, that could indicate higher sensitivity of PSII to photoinhibition (Fig. 4b). Again, similar characteristics have been described for lincomycin-treated leaves (Belgio et al. 2012), which suggests that the mechanisms of NPQ in GL- and WL-acclimated plants (Fig. 4c) differ in their nature: in GL plants a prominent component being the (*F*_0_) quenching of free LHCIIs. In addition, similar Chl fluorescence phenotype was found in plants accumulating geranylgeranylated Chls as reported on transgenic tobacco plants with decreased GGR activity (Grasses et al. 2001) or *Arabidopsis* *lil3* double mutants (*lil3.1*/*lil3.2*) grown under natural white light (Hey et al. 2017), which also documents that accumulation of geranylgeranylated Chls in PPCs after exposure to monochromatic green light is a major factor impairing photosynthetic performance.

### Presence of geranylgeranylated chlorophylls lowers macro-organization of PPCs in the thylakoid membranes

BN-PAGE of thylakoid membranes revealed that, as a result of geranylgeranylated Chls accumulation, the amounts of stable PSII supercomplexes are diminished at the expense of free LHCII trimers and monomers (Fig. 3). This strongly suggests a hampered assembly of PSII-LHCII supercomplexes or their increased degradation in the leaves developed in GL. In order to rule out that this effect is predominantly caused by faster degradation of PSII-LHCII supercomplexes containing geranylgeranylated Chls during solubilization and separation of PPCs from thylakoid membranes, non-invasive spectroscopic methods, CD and 77 K fluorescence spectroscopy were employed to detect the organization of PPCs in the thylakoid membranes.

Amplitudes of two Ψ-type CD bands, the (+) 685 nm and (+) 505, reflecting PPCs organization, were reduced in the thylakoid membranes isolated from GL-acclimated leaves, while the third one at (−) 673 nm remained virtually unchanged in comparison with control plants (Fig. 6a). It is well established that the (+) 685 nm and (−) 673 nm Ψ-type CD bands are associated with Chl chromophores while the (+) 505 Ψ-type CD band mainly originates from a carotenoid (β-carotene) pigment bound to PSII core complexes (Kovács et al. 2006; Tóth et al. 2016). Moreover, the (−) 673 nm band is preferentially associated with grana stacking (Garab et al. 1991), whereas the (+) 505 and (+) 685 nm bands do not depend directly on the granal stacking, but rather on the lateral supramolecular organization of PSII and PSII–LHCII supercomplexes (Tóth et al. 2016; Kovács et al. 2006). Therefore, these results suggest that acclimation to GL leads to a reduced chiral macro-organization of LHCII–PSII supercomplexes, while not affecting the formation of LHCII trimers and granal stacking, which result from subtle interplays of physicochemical forces of repulsion and attraction within chloroplasts with LHCII trimers playing significant roles (Anderson et al. 2008; Chow et al. 2005; Zsiros et al. 2020). This is in line with the electrophoretic data that revealed lower content of LHCII–PSII supercomplexes and, on the contrary, higher content of LHCII unbound to PSII in GL acclimated plants compared to control plants (Fig. 3). This is further supported by the observation that the CD spectrum of GL thylakoids to some extent resemble the features of the LHCII-enriched lincomycin-treated plant leaves (Tóth et al. 2016).

Low temperature spectra of Chl *a* fluorescence emission measured on GL-acclimated thylakoid membranes clearly show that the organization of both PSII and PSI were affected (Fig. 5). GL-acclimated thylakoid revealed pronounced emission at 700 nm originating from LHCII aggregates (Gruszecki et al. 2006; Horton et al. 1991) and the formation of Chl charge transfer states, which have been shown to correlate with the extent of fluorescence quenching (Chmeliov et al. 2016, 2019). These data indicate an impairment of energy transfer between the antenna complex and the core proteins of PSII. Partially quenched, aggregated LHCII were observed, using 77 K steady-state emission spectra and fluorescence lifetime measurements, in the lincomycin-treated plants (Belgio et al. 2012), pointing to the role of hampered formation of LHCII-PSII supercomplexes in GL plants. Further, GL-acclimated thylakoid membranes also revealed greater degree of the dissociation of LHCII trimers to monomers, which possess increased capability of rapid and large quenching (Garab et al. 2002). The shift of PSI emission maximum by 4 nm to shorter wavelengths in GL-acclimated thylakoid membranes can be explained by increased emission from PSI core (F720) at the expense of LHCI emission (F735), which is a sign of PSI-LHCI destabilization (Nellaepalli et al. 2014). Alternative explanation might be that the association of geranylgeranyl Chls with the respective apoproteins affects the structure of the LHCI-PSI supercomplex and cause a blue shift in the 77 K fluorescence emission. To support this notion, it is interesting to point out that Chls dimers in all four Lhca are bound at the inside of LHCI, with their phytol tails protruding into the gap region between LHCI and the PSI core (Qin et al. 2015). Thus, it has been proposed that the interactions between the PSI core and LHCI may be affected by the conformation of the red dimers, especially through the interactions with the hydrophobic phytol tails (Qin et al. 2015; Amunts et al. 2010; Suga et al. 2016).

It is important to note that similar changes of PPCs composition and fluorescence emission spectra as described above were observed with thylakoid membranes incorporating geranylgeranylated Chls in PPCs; as reported on *Arabidopsis* *lil3* double mutants (*lil3.1*/*lil3.2*) (Hey et al. 2017) or cyanobacterium *Synechocystis* sp. PCC 6803 with inactivated GGR (Shpilyov et al. 2005). Also, as pointed out above, LHCII- enriched thylakoid membranes can be obtained by lincomycin treatment of plants, as this antibiotic specifically inhibits protein synthesis in the chloroplast, resulting in the suppression of chloroplast-encoded PS core complexes (Gáspár et al. 2006). Therefore, it is not surprising that thylakoid membranes accumulating geranylgeranylated Chls in their PPCs, which is associated with the loss of PSII core subunits (Hey et al. 2017), show similar features as lincomycin-treated membranes in CD (Tóth et al. 2016) and 77 K spectra, as well as in characteristic features of NPQ (Belgio et al. 2012; Gáspár et al. 2006). These facts indicate that a crucial factor of reduced macro-organization of PPCs in the thylakoid membranes is an excess of LHCII over the PSII core, more or less independently on accumulation of geranylgeranylated Chls. This is supported by the reduced Chl *a*/*b* ratio and the β-carotene content in GL-acclimated thylakoid membranes in comparison with WL ones (Fig. S1a, f). The exact cause of the loss of PSII core subunits associated with geranylgeranylated Chls accumulation is not explained yet, although instability and high vulnerability of PSI and especially PSII to photodegradation could contribute to this effect (Shpilyov et al. 2013). Nevertheless, it is worth noting that phytol chains mediates contact with fatty acids as main interaction between lipid molecules and Chl *a* in PSII core (Loll et al. 2007), hence replacing phytyl with an unsaturated geranylated chain could contribute to PSII core instability.

### Thermal- and photo-stability of PPCs containing geranylgeranylated Chls

As discussed above, instability of PSI and PSII assembled with geranylgeranylated Chls explains the decreased photosynthetic performance and the vulnerability of PPCs in the thylakoid membranes (Shpilyov et al. 2013, 2005; Bollivar et al. 1994). Therefore, we have studied the thermal stability of the PPCs and their macro-organization in thylakoid membranes isolated from GL- and WL-acclimated plants. The thermal stability of the chiral macro-organization of PPCs in the thylakoid membranes was not significantly affected (Fig. 7a–c). Despite the unaltered thermal stability of PPC macro-organization, we observed limited NPQ relaxation in response to strong actinic light in GL-acclimated plants (Fig. 4b), that could indicate higher susceptibility to photoinhibition. This is consistent with earlier observation showing that both PSI and PSII containing geranylgeranyl Chls are unstable and very vulnerable to photodegradation. Increased light sensitivity of both photosystems containing geranylgeranyl Chls was shown in the cyanobacterium *Synechocystis* sp. PCC 6803 with inactivated GGR (Shpilyov et al. 2013, 2005), transgenic tobacco plants with decreased GGR activity (Grasses et al. 2001; Tanaka et al. 1999; Havaux et al. 2003) and rice *chlp* (gene encode GGR) or *lil3* mutant (Li et al. 2019; Zhou et al. 2013). Moreover, the *lil3 chlp* double mutant in rice, exclusively accumulating geranylgeranylated Chl, exhibited lethality at the third-leaf stage (Li et al. 2019), which demonstrates that the complete replacement of phytylated Chls by geranylgeranylated Chls could be fatal to plant survival.

As shown by both CD spectroscopy and CN-PAGE, LHCII trimers of GL plants revealed the most significant decrease in thermal stability (by about 5 °C, Figs. 7d–f and 8); PSII-LHCII supercomplexes as well as PSI-LHCI showed only a slight decrease in the thermal stability, as compared with WL (Fig. 8). The higher thermal susceptibility of LHCII trimers compared to the PSI-LHCI and PSII-LHCII supercomplexes can most probably be accounted for by the higher content of geranylgeranylated Chls in LHCII (cf. Figure 2b, Table S1). It is worth noting that important role on the properties of the LHCII trimers play their interactions with their environment (Akhtar et al. 2019, 2015), when such differences cannot be ruled out in the case of GL- and WL-acclimated plants. Likewise, it was shown recently that rice *chlp* mutant possessed higher sensitivity to high-light stress and an increased lipid peroxidation (Zhou et al. 2013), which may also contribute to changed lipid-protein interactions. However, the transition temperature of thermally induced monomerization of LHCII trimers was also lowered substantially (by 8 °C, in solubilized GL thylakoid membranes compared to the WL control (Fig. 7g–i), suggesting that the instability of LHCII trimers is caused by the presence of geranylgeranylated Chls rather than from altered lipid-protein interactions. Recently, it has been observed that LHCII trimers are stabilized especially by one Chl, which is bound to two different monomers via coordination of the central magnesium ion by histidine of one monomer and hydrophobic interaction of the phytol tail with tryptophan of the other monomer (Seiwert et al. 2017). Unsaturated phytol tail could influence this hydrophobic interaction and thus weaken interactions between adjacent monomers. Therefore, the LHCII trimers containing geranylgeranylated Chls could become particularly sensitive to monomerization.

GGR is also involved in the synthesis of tocopherols preventing the oxidation of membrane lipids triggered by reactive oxygen species and protecting PSII from photoinhibition (Keller et al. 1998; Shibata et al. 2004a; Tanaka et al. 1999), and formation of phylloquinone (vitamin K1) which functions as secondary electron acceptor at the A1 site of PSI (Keller et al. 1998; Shibata et al. 2004b). If the presence of geranylgeranylated Chls in plants cultivated under GL conditions is caused by a deficiency in GGR activity it can be assumed that plants will be deficient in tocopherol and phylloquinone as well, similarly as was observed on GGR mutants (Li et al. 2019; Shpilyov et al. 2013, 2005). Nevertheless, Shpilyov et al. (2013) demonstrated that neither tocopherol nor phylloquinone deficiency in the corresponding mutant can be the cause of the instability and high vulnerability of PSI and especially of PSII to photodegradation. Hence, presence of geranylgeranylated Chls seems to be crucial for susceptibility of both photosystems to high-temperature and photo-oxidative stress.

## Conclusions

Recently, we demonstrated that leaves from various plant species grown under monochromatic green light accumulated a substantial amounts of geranylgeranyl-Chl *a* (Materová et al. 2017). In this study, we demonstrate that all PPCs separated from *Arabidopsis* leaves acclimated to GL incorporated geranylgeranylated Chls *a* and *b*, although their accumulation was preferred in LHCII at the expense of PSII supercomplexes. The presence of geranylgeranylated Chls specifically affected the photosynthetic performance, the macro-organization and the stability of PPCs in the thylakoid membranes. The role of monochromatic green light in determining the deficiency of GGR activity requires further investigations—in particular regarding possible involvement of photoreceptor-mediated acclimation response of plants. These investigations are currently under way in our laboratory.

## Supplementary Information

Below is the link to the electronic supplementary material.

Supplementary file1 (PDF 1431 KB)

## Data Availability

The original contributions presented in the study are included in the article/supplementary material, further inquiries can be directed to the corresponding authors, upon reasonable request.

## Abbreviations

CD: Circular dichroism
Chl: Chlorophyll
CN-PAGE: Clear-native polyacrylamide gel electrophoresis
LHCI: Light-harvesting complex I
LHCII: Light-harvesting complex II
PPC: Pigment-protein complex
PSI: Photosystem I
PSII: Photosystem II
Tm: Transition temperature
Ψ: Polymer and salt induced
