# Supplementary material for: Accumulation of geranylgeranylated chlorophylls in the pigment-protein complexes of Arabidopsis thaliana acclimated to green light: effects on the organization of light-harvesting complex II and photosystem II functions
Source: Photosynth Res. 2021 May 4;149(1-2):233–52. doi: 10.1007/s11120-021-00827-1 (PMC8382614; doi:10.1007/s11120-021-00827-1)
Supplement: Supplementary file 1 — Supplementary file1 (PDF 1431 KB) [file 11120_2021_827_MOESM1_ESM.pdf]

Supplementary Information

Accumulation of geranylgeranylated chlorophylls in the pigment-protein complexes of *Arabidopsis thaliana* acclimated to green light. Effects on the organization of light-harvesting complex II and photosystem II functions

Authors

Václav Karlický[1,2*], Zuzana Kmecová Materová[1], Irena Kurasová[1,2], Jakub Nezval[1], Michal Štroch[1,2], Győző Garab[1,3*], Vladimír Špunda[1,2*]

Affiliation

[1]Department of Physics, Faculty of Science, University of Ostrava, Chittussiho 10, CZ-710 00 Ostrava, Czech Republic

[2]Global Change Research Institute, Czech Academy of Sciences, Bělidla 986/4a, 603 00 Brno, Czech Republic

[3]Institute of Plant Biology, Biological Research Center, Temesvári körút 62, H-6726 Szeged, Hungary

***Corresponding Authors:** vaclav.karlicky@osu.cz, garab.gyozo@brc.hu, vladimir.spunda@osu.cz

Table S1: Contents of geranylgeranylated Chls in different PPCs of A. thaliana plants acclimated to green light, relative to the total amounts found in the thylakoid membranes. *Abundances of geranylgeranylated Chls a and b (sum of THGG, DHGG and GG Chls) in different PPCs were normalized to the amounts in the thylakoid membranes (TM) from which the separation was performed. In the thylakoid membranes, the geranylgeranylated Chls a and b were present at 53.3 ± 3.8 % and 58.6 ± 4.6 %, respectively. The data are means of three independent GL-acclimation ± SD. The thylakoid membranes were isolated from newly developed leaves of green-light acclimated plants. Data within a row followed by the same letter are not significantly different (one-factor ANOVA followed by Tukey's post hoc test with significance level at P<0.05).*

	TM	C2S2M2	C2S2M	C2S2	C2S	PSI+C2	LHCII assembly	LHCII trimers	LHCII momomers
Chl *a*	100	84.7 ± 4.2^{a}	87.8 ± 5.9^{a}	90.1 ± 3.4^{ab}	90.0 ± 5.0^{ab}	89.1 ± 9.2^{ab}	100.0 ± 7.5^{bc}	103.2 ± 4.7^{c}	109.1 ± 6.2^{c}
Chl *b*	100	78.8 ± 4.3^{a}	81.5 ± 10.8^{ab}	83.7 ± 4.2^{ab}	90.9 ± 2.8^{b}	91.6 ± 9.7^{b}	90.4 ± 5.0^{ab}	90.2 ± 7.2^{ab}	111.3 ± 6.8^{c}

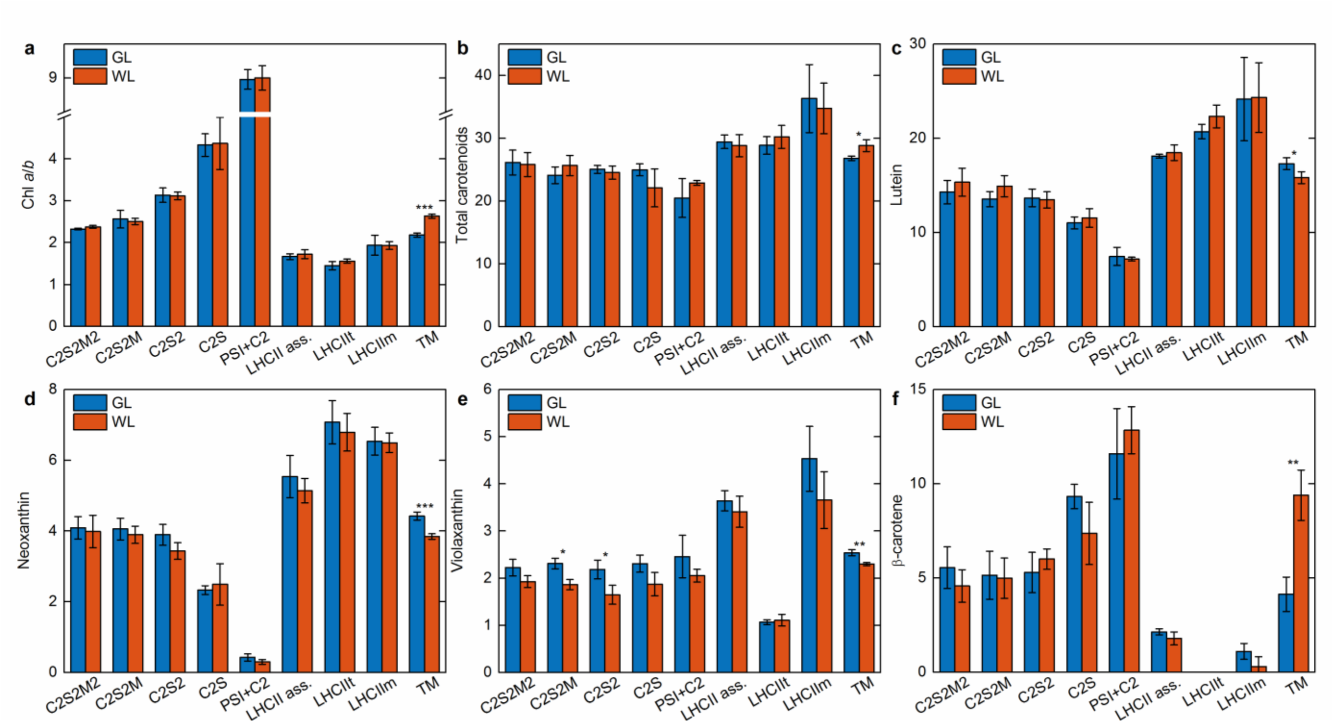

Fig. S1: Pigment composition of the PPCs and thylakoid membranes isolated from newly developed leaves of Arabidopsis acclimated to green (GL) and white (WL) lights. *The contents of individual carotenoids were normalized to 100 total Chls. Mean values and standard errors from 3 independent experiments are displayed. Asterisks indicate statistically significant differences (Student's t-test) between GL and WL (*, P < 0.05; **, P < 0.01; ***, P < 0.001).*

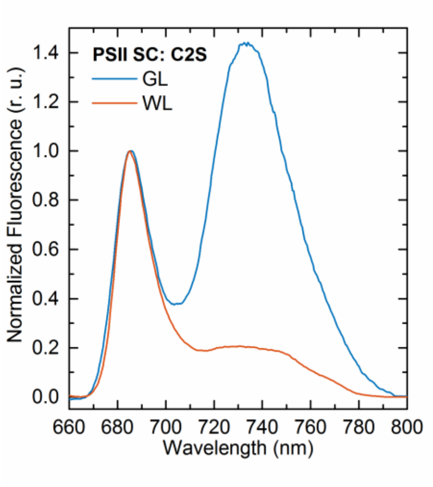

Fig. S2: Chl a fluorescence spectra of band designated as C2S at 77 K. *Typical spectra of the smallest PSII supercomplexes (PSII SC) C2S band separated by CN-PAGE from thylakoid membranes isolated from Arabidopsis acclimated to green (GL) and white (WL) light; spectra were measured on the green gel pieces cut from gel shown in Fig. 2; samples were excited at 476 nm (preferential excitation of Chl b); the spectra are normalized on PSII emission.*

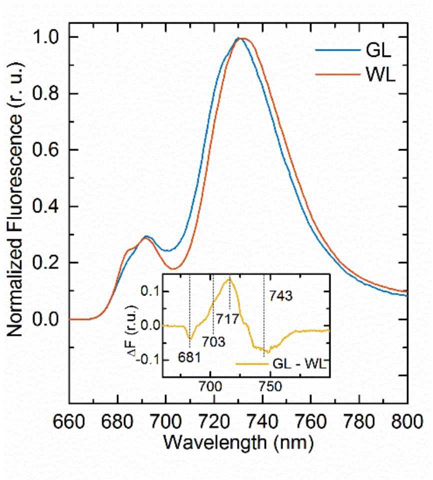

Fig. S3: Chl a fluorescence spectra of intact leaves at 77 K. *Average spectra from six independent experiments on intact Arabidopsis leaves acclimated to green (GL) and white (WL) light; excitation wavelength, 476 nm (preferential excitation of Chl b); the spectra are normalized on PSI emission; inset, GL-WL difference spectrum.*

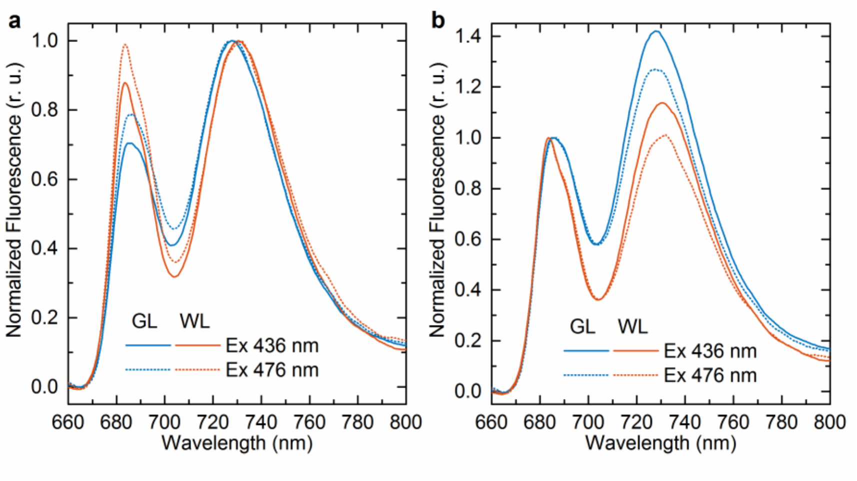

Fig. S4: Comparison of 77 K Chl a fluorescence spectra of Arabidopsis thylakoid membranes isolated from dark-adapted leaves acclimated to green (GL) and white (WL) light, excited at preferential excitation of Chl a (436 nm) and Chl b (476 nm). *The spectra are normalized to (a) PSI and (b) PSII emission. Average spectra from four independent experiments.*

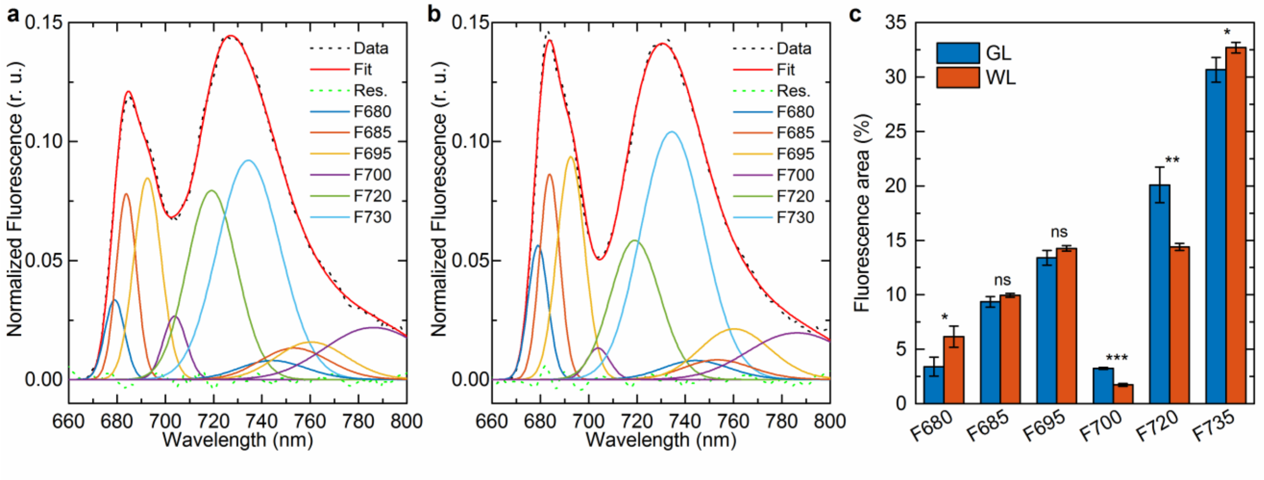

Fig. S5: Gaussian decomposition of the 77 K Chl a fluorescence emission spectra of thylakoid membranes. *Typical fluorescence emission spectra with Gaussian decompositions of thylakoids from leaves acclimated to green (a) and white (b) light. The spectra are normalized on the same total (integrated) fluorescence intensity. The six main components are identified as F680 (peak 681 nm), F685 (peak 685 nm), F695 (peak 693 nm), F700 (peak 700 nm), F720 (peak 720 nm), and F735 (peak 735 nm). They correspond to the fluorescence maxima of the trimeric and monomeric forms of LHCII, the core antenna complex of PSII (CP43 – F685, CP47 - F695), the aggregated trimers of LHCII, the core complex of PSI, and LHCI, respectively. (c) Integrated areas of the fluorescence intensities of the PPCs (emission bands) of thylakoid membranes at 77 K, spectra excited at 476 nm, estimated from the decomposed fluorescence emission spectra. The data are means of four independent measurements ± S.D. Asterisks indicate statistically significant difference (Student's t-test) between GL and WL (*, P < 0.05; **, P < 0.01; ***, P < 0.001).*

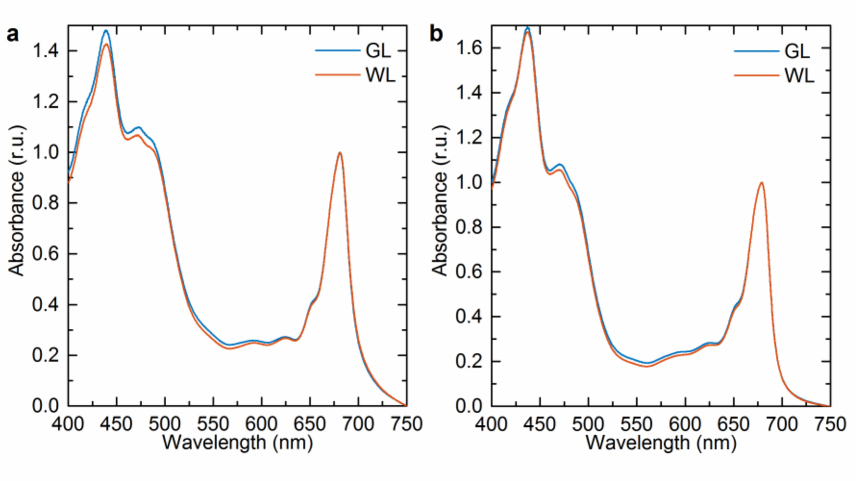

Fig. S6: Absorption spectra. *Average spectra from four independent experiments of thylakoid membranes isolated from dark adapted Arabidopsis plants acclimated to green (GL) and white (WL) light. The thylakoid membranes were isolated from newly developed leaves grown in GL and WL, and were measured in stacked (a) and unstacked (b) states. The spectra are normalized to the* Q_y *absorbance maxima.*

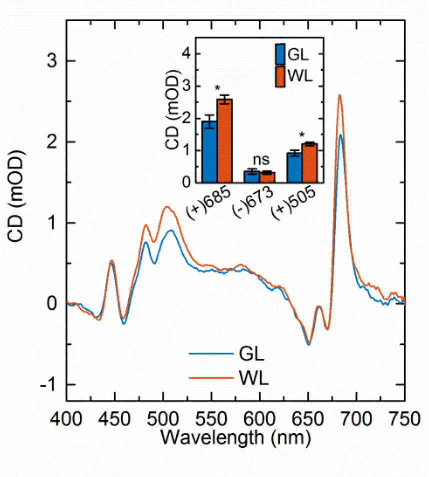

Fig. S7: CD spectra of intact leaves. *Average spectra from six independent experiments measured on detached leaves of Arabidopsis plants acclimated to green (GL) and white (WL) light. Inset displays the amplitudes of the three* Ψ*-type CD bands at (+)685, (-)673 and (+)505 nm, with reference wavelengths at 750, 620 and 620 nm, respectively; mean values ± SD (n = 6). Asterisks indicate statistically significant differences (Student's t-test) between GL and WL (*, P < 0.05; **, P < 0.01; ***, P < 0.001).*
